# Construction and Validation of a Combined Ferroptosis and Hypoxia Prognostic Signature for Hepatocellular Carcinoma

**DOI:** 10.3389/fmolb.2021.809672

**Published:** 2021-12-17

**Authors:** Kai Wen, Yongcong Yan, Juanyi Shi, Lei Hu, Weidong Wang, Hao Liao, Huoming Li, Yue Zhu, Kai Mao, Zhiyu Xiao

**Affiliations:** ^1^ Department of Hepatobiliary Surgery, Sun Yat-Sen Memorial Hospital, Sun Yat-Sen University, Guangzhou, China; ^2^ Department of Pathology, The Seventh Affiliated Hospital, Sun Yat-Sen University, Shenzhen, China; ^3^ Department of Thyroid Surgery, Sun Yat-Sen Memorial Hospital, Sun Yat-Sen University, Guangzhou, China

**Keywords:** hepatocellular carcinoma, ferroptosis, hypoxia, immune microenvironment, gene signature, nomogram

## Abstract

**Background:** Ferroptosis, as a unique programmed cell death modality, has been found to be closely related to the occurrence and development of hepatocellular carcinoma (HCC). Hypoxia signaling pathway has been found to be extensively involved in the transformation and growth of HCC and to inhibit anti-tumor therapy through various approaches. However, there is no high-throughput study to explore the potential link between ferroptosis and hypoxia, as well as their combined effect on the prognosis of HCC.

**Methods:** We included 370 patients in The Cancer Genome Atlas (TCGA) database and 231 patients in the International Cancer Genome Consortium (ICGC) database. Univariate COX regression and Least Absolute Shrinkage and Selection Operator approach were used to construct ferroptosis-related genes (FRGs) and hypoxia-related genes (HRGs) prognostic signature (FHPS). Kaplan–Meier method and Receiver Operating Characteristic curves were analyzed to evaluate the predictive capability of FHPS. CIBERSOR and single-sample Gene Set Enrichment Analysis were used to explore the connection between FHPS and tumor immune microenvironment. Immunohistochemical staining was used to compare the protein expression of prognostic FRGs and HRGs between normal liver tissue and HCC tissue. In addition, the nomogram was established to facilitate the clinical application of FHPS.

**Results:** Ten FRGs and HRGs were used to establish the FHPS. We found consistent results in the TCGA training cohort, as well as in the independent ICGC validation cohort, that patients in the high-FHPS subgroup had advanced tumor staging, shorter survival time, and higher mortality. Moreover, patients in the high-FHPS subgroup showed ferroptosis suppressive, high hypoxia, and immunosuppression status. Finally, the nomogram showed a strong prognostic capability to predict overall survival (OS) for HCC patients.

**Conclusion:** We developed a novel prognostic signature combining ferroptosis and hypoxia to predict OS, ferroptosis, hypoxia, and immune status, which provides a new idea for individualized treatment of HCC patients.

## Introduction

Epidemiological studies have shown that the incidence of liver cancer is the sixth most common worldwide. Hepatocellular carcinoma (HCC) is the most common type, which has the characteristics of high recurrence rate and poor prognosis (Llovet et al., 2021; Sung et al., 2021). Surgical treatment (including hepatectomy and liver transplantation) is the primary treatment for HCC, however, due to early HCC patients having no obvious symptoms, the majority of HCC patients are diagnosed at an advanced stage without the chance of surgery (European Association for the Study of the Liver et al., 2018; Marrero et al., 2018). Lenvatinib and sorafenib are the first-line agents for advanced HCC, with a median survival time of 12.3 versus 13.6 months (Llovet et al., 2021). The emergence of immunotherapy has brought new light to the treatment of HCC. At present, immune checkpoints inhibitors (ICIs) monotherapy (nivolumab, pembrolizumab, camrelizumab, etc.) is mainly used for the second-line treatment of advanced HCC and several relevant clinical trials are being carried out (El-Khoueiry et al., 2017; Llovet et al., 2018; Qin et al., 2020). However, although there are many options of treatment for advanced HCC, only a fraction of patients can benefit from it in clinical practice. Therefore, there is an urgent requirement to develop a potential signature to classify HCC patients for individualized treatment.

Ferroptosis, a newly discovered pattern of regulatory cell death, is different from cell programmed necrosis, apoptosis, and autophagy, and is a result of iron-dependent lipid peroxidation and the accumulation of reactive oxygen species (Stockwell et al., 2017). In recent years, inducing ferroptosis in tumor cells has become a promising treatment modality. For example, sorafenib could inhibit cystine-glutamate antiporter and leads to glutathione depletion, which induces ferroptosis in HCC cells (Hassannia et al., 2019; Liang et al., 2019). Notably, Louandre et al. found that inactivation of retinoblastoma (Rb) may contribute to the antitumor effect of sorafenib on HCC by sensitizing HCC cells to ferroptosis (Louandre et al., 2015). However, the underlying mechanism still needs to be elucidated. As research on ferroptosis progressed, many genes have been confirmed as drivers of ferroptosis (DOFs) and suppressors of ferroptosis (SOFs). The effect of these ferroptosis-related genes (FRGs) on tumor immunity, tumor microenvironment (TME), and prognosis of HCC patients has not been clarified.

Hypoxia, widely present in various solid tumors, especially HCC, is due to the large consumption of oxygen by tumors during proliferation and breaking the balance between oxygen supply and consumption, which eventually produces a hypoxia microenvironment (Menrad et al., 2010). Hypoxia can stimulate angiogenesis, promote cell proliferation, inhibit cell differentiation and apoptosis, and accelerate the metastasis and invasion of HCC (Gwak et al., 2005; Piret et al., 2005). In addition, multiple studies have shown that hypoxia interacts with immune cells such as tumor-associated macrophages (TAMs), myeloid-derived suppressor cells (MDSCs), and regulatory T cells (Tregs) to promote immunosuppression of tumor cells and accelerate tumor cell immune escape (Corzo et al., 2010; Wang et al., 2018; Riera-Domingo et al., 2020). And the interaction of tumor immune microenvironment and immune cells has been confirmed to be a critical factor in HCC progression as well as response to immunotherapy (Cariani and Missale, 2019). These studies suggest that hypoxia may play a central role in the immunotherapy of HCC.

Notably, a recent study indicated that hypoxia-inducible factor 1 subunit α (HIF1A) could affect lipid metabolism and store lipids in droplets, thereby reducing peroxidation-mediated endomembrane damage, which could limit ferroptosis in cells (Yang et al., 2019). And another study showed that HIF-2α could activate the expression of HILPDA and selectively enrich polyunsaturated lipids, which could promote ferroptosis in cells (Zou et al., 2019). These results suggest that there may be some potential associations between ferroptosis and hypoxia, and the effect of the interaction between ferroptosis and hypoxia on HCC prognosis needs further exploration.

In this study, we used the International Cancer Genome Consortium (ICGC) and The Cancer Genome Atlas (TCGA) databases, combined with known FRGs and hypoxia related genes (HRGs), to construct a prognostic signature based on FRGs and HRGs, which is significantly associated with HCC prognosis, ferroptosis status, hypoxia status, and immune status.

## Material and Methods

### Data Acquisition

The mRNA expression data [level 3; Fragment Per Kilobase Million (FPKM) normalized] and corresponding clinicopathological information of HCC patients were obtained from the TCGA^1^ and ICGC^2^ websites. A total of 370 HCC patients from TCGA database were enrolled in the training cohort, and 231 patients from the ICGC database were enrolled in the validation cohort, after excluding patients who lacked important clinical information. From the FerrFb database^3^ and previous publications, 227 FRGs were retrieved. 200 HRGs were downloaded from Molecular Signatures Database^3^ as other studies have reported. Gene expression data from both databases were normalized by the R package “limma”. Table 1 showed the clinicopathological information of each cohort in this study.

**TABLE 1:** Clinicopathological features of the samples included in this study.

	TCGA		ICGC	
	Number	Percentage	Number	Percentage
Total	370	100.00%	231	100.00%
Age	16-90 (61)		31-89 (69)	
<median	177	47.84%	115	49.78%
≥median	193	52.16%	116	50.22%
Gender				
Male	249	67.93%	170	73.59%
Female	121	32.07%	61	26.41%
AFP				
<400	213	57.57%	−	−
≥400	64	17.30%	−	−
NA	93	25.13%		
WHO Grade				
I-II	232	62.70%	−	−
III-IV	133	35.95%	−	−
NA	5	1.35%		
TNM Stage				
I-II	256	69.19%	141	61.04%
III-IV	90	24.32%	90	38.96%
NA	24	6.49%	−	−

### Development of the FHPS

Univariate COX regression analysis was used to filtrate FRGs and HRGs associated with prognosis in the TCGA cohort. Thereafter, the FRGs and HRGs significantly associated with prognosis (*p* < 0.0005) of HCC patients were input into the Least Absolute Shrinkage and Selection Operator (LASSO) COX regression model to identify the critical genes and the corresponding regression coefficient by using the R package “glmnet” (Friedman et al., 2010). We constructed a FRGs and HRGs prognostic signature (FHPS) for the HCC patients involving 10 FRGs and HRGs. FHPS scores were calculated for all patients according to the formula:

$$FHPS\;score=\overset{n}{\underset{i=1}{\sum }}Coef_{i}\text{*}x_{i}$$

Where $x_{i}$ is the FPKM value of each FRG or HRG and $\;Coef_{i}$ is the coefficient.

The FHPS score of all HCC patients was calculated based on the above formula. Then R package “survminer” was used to calculate the optimal cut-off value (this is an outcome-oriented method providing a value of a cut-off point that corresponds to the most significant relation with survival^4^) and the patients were divided into two subgroups (low-FHPS and high-FHPS groups) according to the optimal cut-off value. PCA analysis was performed using R software and scatter diagrams were plotted using the R package “ggplot2” in both databases.

### Functional Analysis

Gene Set Enrichment Analysis (GSEA) was used to investigate the pathways enriched in low-FHPS subgroup and high-FHPS subgroup. Pathways that satisfy the following conditions are defined as significant enrichment pathways: normalized enrichment score > 1, nominal *p* < 0.05, and false discovery rate q < 0.25.

Differentially expressed genes (DEGs) between the high-FHPS and low-FHPS groups were obtained using R package “limma” (| log2(Fold change) | > 1 and adjust *p* < 0.05). Then DEGs were input into Metascape^5^ for functional enrichment and pathway analysis, including Kyoto Encyclopedia of Genes and Genomes Pathway (KEGG pathway) and Gene Ontology (GO) analysis.

The FRGs and HRGs significantly associated with prognosis (*p* < 0.0005) of HCC patients were input into the Search Tool for the Retrieval of Interacting Genes website to develop a protein–protein interaction (PPI) network. And Pearson method was used to construct correlation network of prognostic FRGs and HRGs.

Simple nucleotide variation data of HCC patients in TCGA cohort were obtained from TCGA website and the R package “maftools” was used to analyze the differences in genomic alterations between the low-FHPS and high-FHPS groups.

### Analysis of Immune Cell Infiltration

In order to investigate the difference of immune infiltration status between patients in the high-FHPS group and low-FHPS group, single-sample GSEA (ssGSEA) was used to calculate the infiltration of 28 immune cells in the tumor immune microenvironment (TIME) of HCC patients using the R package “GSVA”. Moreover, we also use EPIC and CIBERSORT methods to calculate immune cell characteristics for HCC patients.

### Analysis of the Protein Expression of Prognostic FRGs or HRGs Between in Normal Liver Tissue and HCC Tissue by Immunohistochemistry

We collected nine pairs of HCC tissues and adjacent normal liver tissues from Sun Yat-Sen Memorial Hospital, Sun Yat-Sen University which was approved by the ethics committees of Sun Yat-Sen Memorial Hospital, Sun Yat-Sen University. The HCC tissues and adjacent normal liver tissues were fixed with 10% formalin, embedded by paraffin, and sectioned. Then we selected the optimal tissue sections for degreasing and Immunohistochemistry staining. Antibodies used in this study are as follows: PPARGC1A (Proteintech, 66369-1-IG), SLC7A11 (Proteintech, 26864-1-AP). In addition, immunohistochemical staining images of the remaining eight prognostic ferroptosis-related IRGs were obtained from the Human Protein Atlas^6^ (HPA). The number of tissues and corresponding clinicopathological information is shown in Supplementary Table S1.

### Statistical Analysis

The overall survival (OS) of different subgroups (Including high- and low-FHPS subgroups, and additional subgroups) were compared using the Kaplan–Meier method and the log-rank test. The chi-square test was used to compare the clinical characteristics of the low-FHPS and high-FHPS groups (Table 2). Multivariate and univariate COX regression was performed to determine that the predictive power of FHPS was independent of other clinical characteristics in HCC patients.

**TABLE 2:** Clinicopathological features between low- and high-FHPS groups.

	TCGA		CGGA			
	Low-FHPS	High-FHPS	p value	Low-FHPS	High-FHPS	p value
Total case	164	206		171	60	
Age			0.469	0.575		
<median	75	102		87	28	
≥median	89	104		84	32	
Gender			0.888	0.958		
Male	111	138		126	44	
Female	53	68		45	16	
AFP			0.044	−		
<400	107	106		−	−	
≥400	23	41		−	−	
NA	34	59				
WHO Grade			<0.001	−		
I-II	122	110		−	−	
III-IV	38	95		−	−	
NA	4	1				
TNM Stage			<0.001			<0.001
I-II	129	127		118	23	
III-IV	22	68		53	37	
NA	13	11				

Independent risk factors obtained by multivariate COX regression analysis were used to construct a nomogram to predict OS for HCC patients. Then, the nomogram was verified by the calibration curve and the C-index both in the ICGC and TCGA cohorts. The above construction and validation of nomogram was performed using package “rms”. In addition, Receiver Operating Characteristic (ROC) curve was used to compare the predictive accuracy of nomogram with other prognostic factors using R package “timeROC”.

Statistical analysis in this study was performed using IBM SPSS Statistics (version 25.0) and the R software (version 4.1.0).

## Results

### Construction of the FHPS in the TCGA Cohort

**FIGURE 1:**
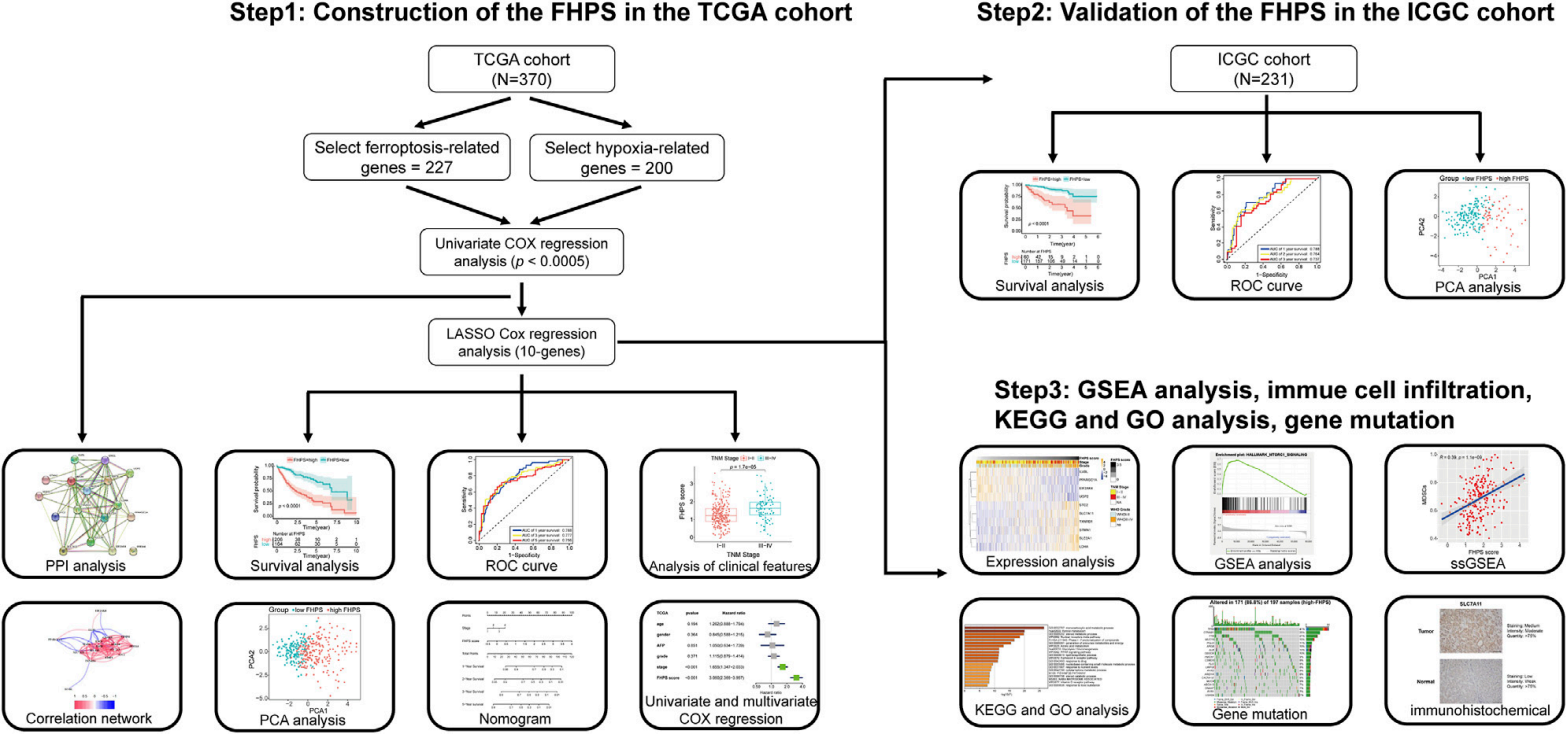
Study flow chart.

**FIGURE 2:**
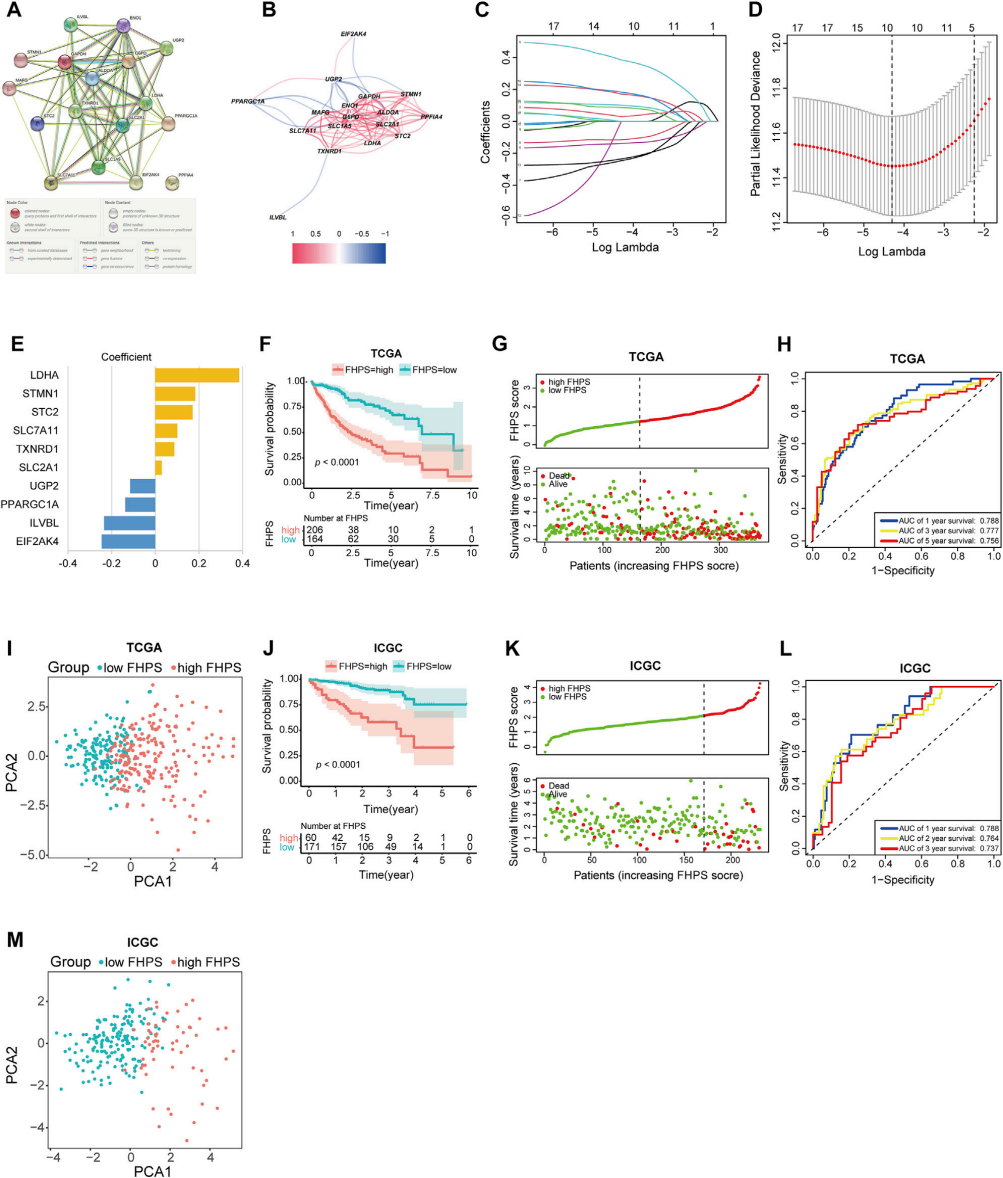
**(A, B)** The PPI and correlation network suggesting the relationship between prognostic FRGs and HRGs. **(C–E)** LASSO regression was performed, calculating the crucial genes **(C, D)** and coefficients **(E)**. **(F)** Kaplan–Meier curves showed that the high-FHPS group had worse overall survival than the low-FHPS group in TCGA cohort. **(G)** Distributions of FHPS scores and survival status of HCC patients in the TCGA cohort. **(H)** ROC curves of FHPS for predicting the 1/3/5-years survival in the TCGA cohort. **(I)** The plots of the PCA of the TCGA cohort based on the expression profiles of the 10 signature genes in different FHPS groups. **(J)** Kaplan–Meier curves showing that the high-FHPS group had worse overall survival than the low-FHPS group in the ICGC cohort. **(K)** Distributions of FHPS scores and survival status of HCC patients in the ICGC cohort. **(L)** ROC curves of FHPS for predicting 1/2/3-years survival in the ICGC cohort. **(M)** The plots of the PCA of the ICGC cohort based on the expression profiles of the 10 signature genes in different FHPS groups.

The detailed flow chart is shown in Figure 1. Univariate COX regression was used to screen for ferroptosis-related prognostic genes or hypoxia-related prognostic genes (FHRPGs) in the TCGA cohort. In the condition of *p* < 0.0005, there were 17 FHRPGs significantly associated with the prognosis of HCC patients (Table 3). The PPI and correlation network of 17 FRGs and HRGs indicated that there was a strong correlation between the FRGs and HRGs (Figures 2A,B). Subsequently, 17 FHRPGs were entered into the LASSO Cox regression analysis, resulting in a 10-FHRPGs prognostic signature, with LDHA, PPARGC1A, UGP2, ILVBL and STC2 as HRGs, EIF2AK4, SLC7A11, TXNRD1 and STMN1 as FRGs, and SLC2A1 as both HRG and FRG (Figures 2C–E). FHPS scores for HCC patients in TCGA cohort were calculated according to the formula, and the patients were divided into high-FHPS group (206 HCC patients) and low FHPS group (164 HCC patients) according to the optimal cut-off value. Kaplan-Meier curves and the log-rank test showed that patients in the high-FHPS group had poor outcomes (*p* < 0.001), including a higher incidence of death and a shorter survival time (Figure 2F). The distributions of survival status and FHPS score are shown in Figure 2G and HCC patients with higher FHPS scores had shorter overall survival and higher mortality. As shown in Figure 2H, FHPS has a strong predictive ability for OS of HCC patients (The Area Under Curve (AUC) of 1-, 3-, and 5-years reached 0.788, 0.777, and 0.756). PCA revealed that the HCC patients in low-FHPS and high-FHPS groups were distinctively clustered (Figure 2I).

**TABLE 3:** The seventeen ferroptosis-related genes and hypoxia-related genes.

id	HR	HR.95L	HR.95H	pvalue
G6PD	1.410486192	1.247924793	1.594223715	3.69196E-08
SLC1A5	1.329390229	1.176100654	1.502659128	5.2423E-06
EIF2AK4	0.448475019	0.302313364	0.66530252	6.74369E-05
SLC7A11	1.380676705	1.161742683	1.640869523	0.000250371
TXNRD1	1.34537543	1.142091441	1.584842494	0.000385801
STMN1	1.363925798	1.148365361	1.61994923	0.000406068
MAFG	1.66871613	1.256259604	2.216590833	0.00040796
ENO1	1.539529538	1.284776167	1.844796984	2.93895E-06
LDHA	1.848256606	1.410815639	2.421331594	8.28705E-06
PPARGC1A	0.71815381	0.619369171	0.832693842	1.16036E-05
UGP2	0.6432189	0.515202649	0.803044304	9.73323E-05
GAPDH	1.363480486	1.157305827	1.606385272	0.000210128
ALDOA	1.330054995	1.142683761	1.548150371	0.000231744
PPFIA4	3.534285474	1.793991564	6.962782915	0.000262952
ILVBL	0.626582736	0.48588439	0.808023334	0.000314786
STC2	1.398931697	1.164666714	1.680317527	0.000330512
SLC2A1	1.559522656	1.312872076	1.852511726	4.21491E-07

Genes without shaded were FRGs, light grey shade are HRGs, grey shaded were both HRG, and FRG.

### Validation of the FHPS in the ICGC Cohort

FHPS scores were calculated for all patients in the ICGC cohort using the same formula, and the patients were divided into low-FHPS group (171 HCC patients) and high-FHPS group (60 HCC patients) according to the optimal cut-off value. The results of ICGC cohort are mostly consistent with TCGA cohort; patients in the high-FHPS group had lower survival rates and shorter survival (Figure 2J). The distribution of survival status and FHPS scores also indicated that patients with higher FHPS scores had shorter overall survival time and higher mortality (Figure 2K). The ROC curve showed that FHPS score also had strong predictive power in the ICGC cohort. The AUCs were: 0.788 (1-year), 0.764 (2-years), and 0.737 (3-years) (Figure 2L). Due to the small number of patients with OS over 5 years in ICGC database (only two cases), the 5-years AUC value was not calculated in ICGC cohort in order to reduce bias. PCA analysis in the ICGC cohort also showed significant differences in the distribution of patients between the two groups. (Figure 2M). These results indicated that the FHPS score can accurately predict the prognosis of HCC patients.

### Prognostic Analysis of the 10 FHRPGs

**FIGURE 3:**
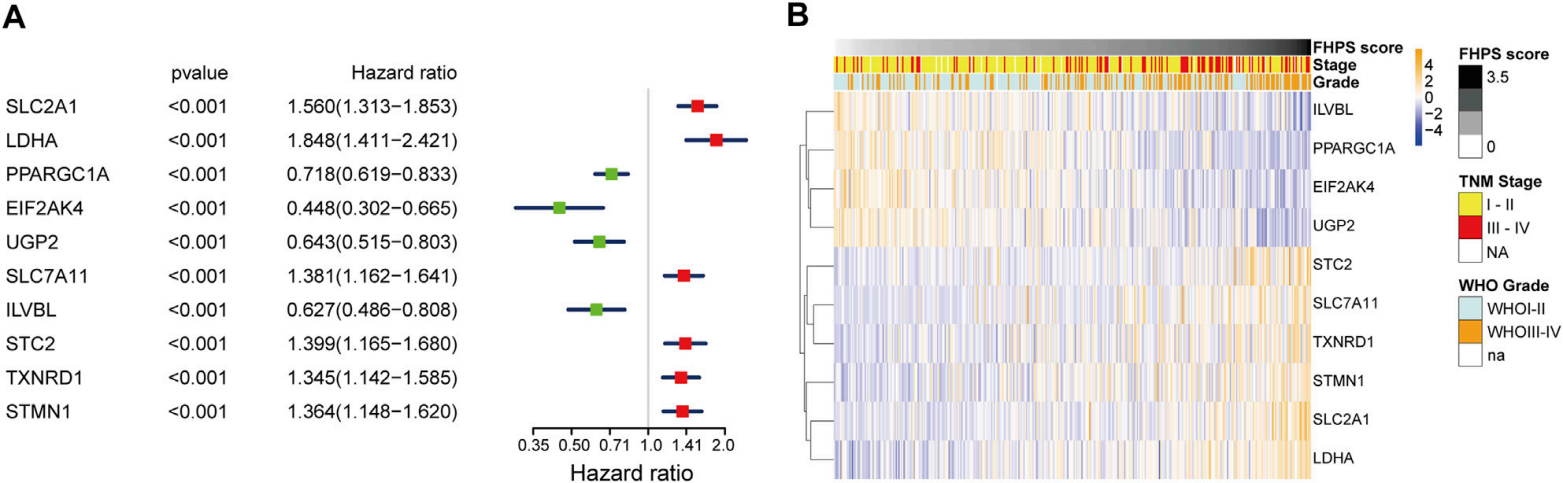
**(A)** Forest plot of the prognostic ability of the 10 signature genes. **(B)** Heatmap of the associations between the expression levels of the 10 signature genes and clinicopathological features in the TCGA cohort.

Univariate COX regression analysis of 10 FHRPGs showed that PPARGC1A, EIF2AK4, UGP2, and ILVBL were protective factors in HCC patients [Hazard Ratio (HR) < 1; *p* < 0.001] while SLC2A1, LDHA, SLC7A11, STC2, TXNRD1, and STMN1 were risk factors in HCC patients (HR > 1; *p* < 0.001) (Figure 3A). In addition, the heatmap indicated that the expression of 10 FHRPGs was significantly different between the high-FHPS group and the low-FHPS group. The expressions of PPARGC1A, EIF2AK4, UGP2, and ILVBL decreased with the increase of FHPS score while the expression of SLC2A1, LDHA, SLC7A11, STC2, TXNRD1, and STMN1 increased with the increase of FHPS score. In addition, their expression level is also related to WHO grade and TNM Stage of HCC patients (Figure 3B).

### Stratification Analysis of the FHPS

**FIGURE 4:**
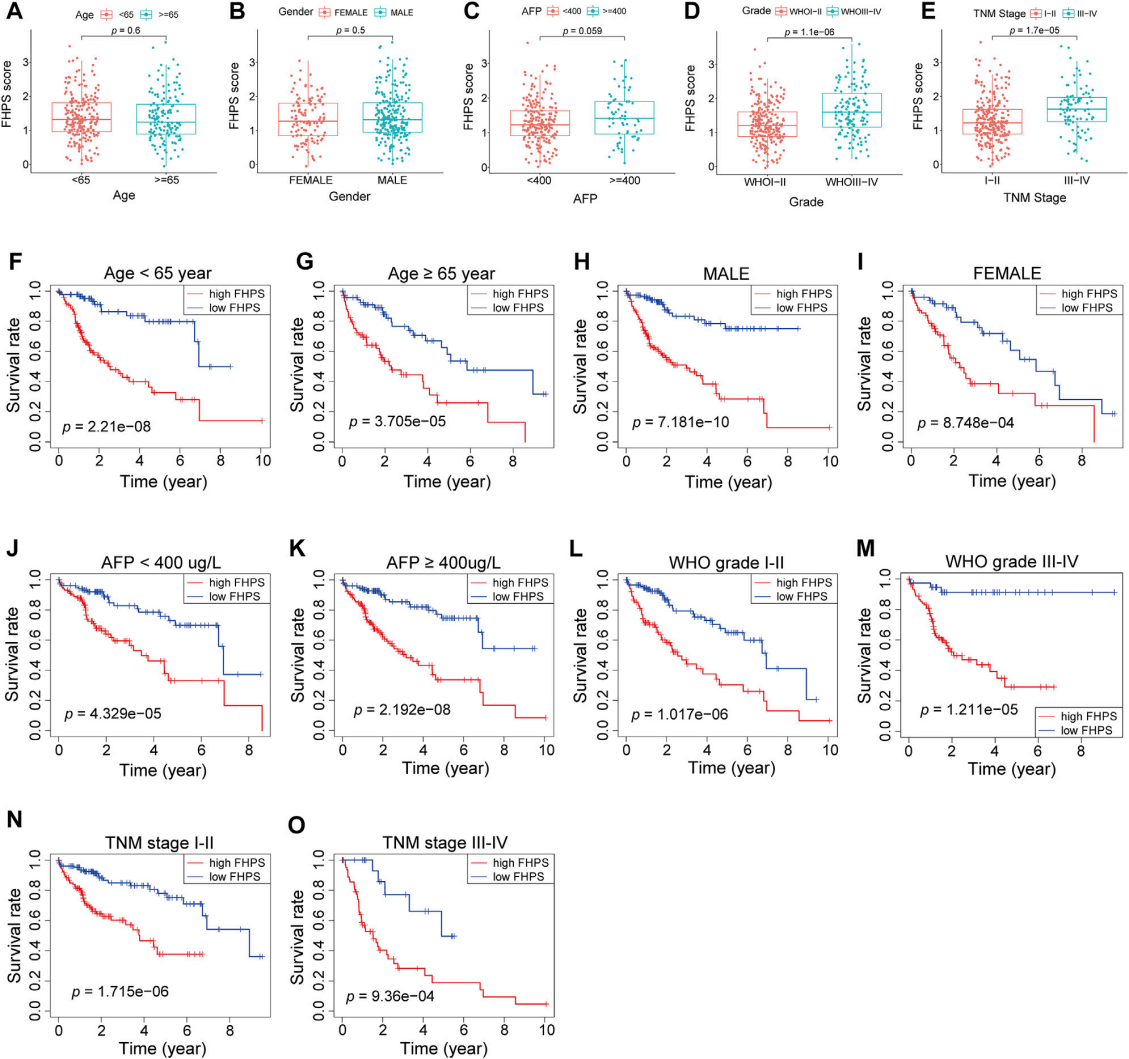
**(A–E)** Patients with different clinicopathological features (including WHO grade and TNM stage, but not AFP value, age, and gender) had different levels of FHPS scores. **(F–O)** The FHPS retained its prognostic value in multiple subgroups of HCC patients (including patients with WHO grade III-IV or I-II, TNM stage III-IV or I-II, male or female, age <65 or ≥ 65 years and AFP < 400 or ≥ 400 ug/ L).

To investigate whether the FHPS score was associated with clinicopathological characteristics of HCC patients, we analyzed the FHPS score of HCC patients contrasting different clinical subgroups, as shown in Figures 4A–E: HCC patients with WHO grade III-IV and TNM stage III-IV had higher FHPS score. While the distribution of FHPS score were not associated with age, gender, α-fetoprotein (AFP), < or >= 400 ug/ L. In addition, to investigate the prognostic power of FHPS in different subgroups of HCC patients, we performed survival analysis in multiple clinical subgroups. The Kaplan–Meier curves showed that FHPS score also had strong predictive power in different clinical subgroups. In TNM I-II and TNM III-IV subgroups, HCC patients with low-FHPS had better OS compared with HCC patients with high-FHPS, and the same results were obtained for whoI-II and WHOIII-IV, AFP < 400 and >= 400 ug/ L, female and male, age < 65 and age >= 65 subgroups (Figures 4F–O). The above results indicate that the FHPS score we developed has a stable ability to predict prognosis for HCC patients in various clinical subgroups.

### Analysis of Ferroptosis and Hypoxia Status

**FIGURE 5:**
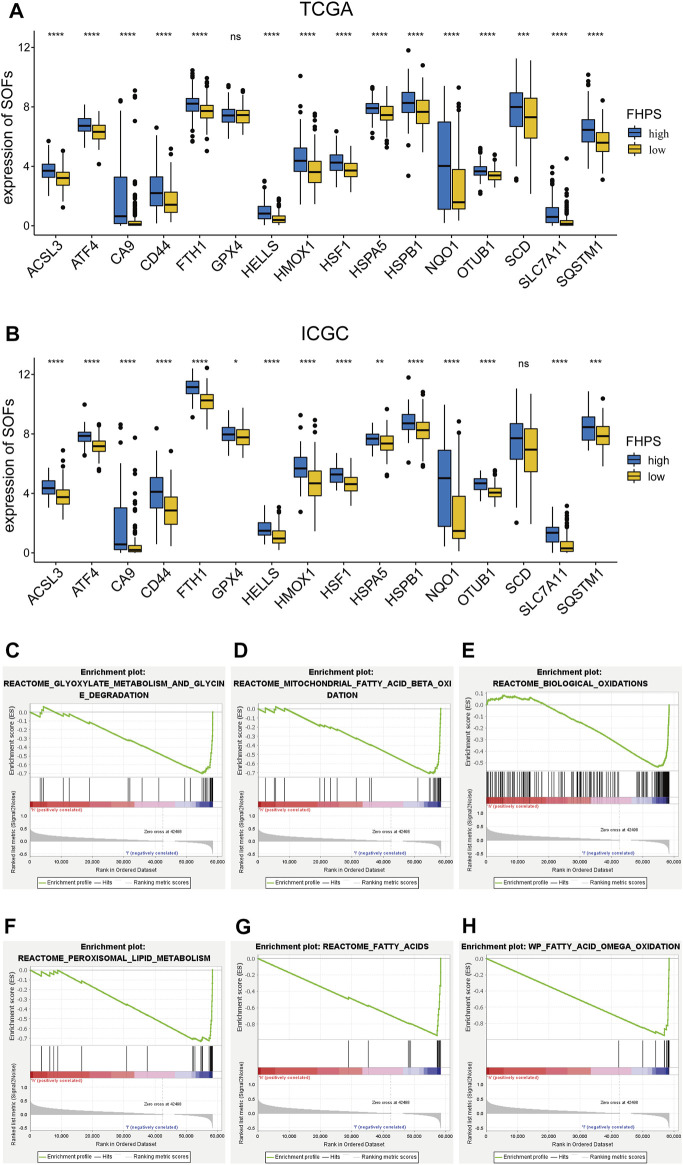
**(A, B)** Comparison of the expressions of the suppressors of ferroptosis between the high- and low-FHPS groups of the **(A)** TCGA and **(B)** ICGC cohorts. **(C–H)** Gene set enrichment analysis (GSEA) (Ferroptosis-related pathways) of the high-FHPS and low-FHPS subgroups in the TCGA cohorts. *p < 0.05; **p < 0.01; ***p < 0.001; ****p < 0.0001.

To investigate the association of FHPS with ferroptosis status in HCC patients, we first used *t*-test to compare the expression levels of SOFs and DOFs in high-FHPS and low-FHPS groups. ACSL3, ATF4, CA9, CD44, FTH1, GPX4, HELLS, HMOX1, HSF1, HSPA5, HSPB1, NQO1, OTUB1, SCD, SLC7A11, and SQSTM1 are well-researched SOFs. As shown in Figure 5A, in the TCGA cohort, except for GPX4, the expression of the remaining SOFs were upregulated in the high-FHPS group. We validated this result in the ICGC cohort and showed that, like the TCGA cohort, the majority of the SOFs (ACSL3, ATF4, CA9, CD44, FTH1, GPX4, HELLS, HMOX1, HSF1, HSPA5, HSPB1, NQO1, OTUB1, SLC7A11, and SQSTM1) were significantly upregulated in the high-FHPS group (Figure 5B). In addition, we also compared the expression of DOFs between the two groups in TCGA cohort and ICGC cohort. The results showed that in the low-FHPS group, about half of the DOFs (ALOX12, ALOX15, ANO6, ATF3, ATG5, ATG7, DPP4, EGFR, ELAVL1, HMGB1, IREB2, KEAP1, PEBP1, SAT1 and ZEB1 in TCGA cohort; ALOX12, ANO6, ATM, BAP1, DPP4, EGFR, IDH1, IREB2, NCOA4, PEBP1, and ZEB1 in ICGC cohort) were up-regulated (Supplementary Figures S1A,B). These results suggest that ferroptosis might be more significant in the low-FHPS group, whereas the ferroptosis status of patients in the high-FHPS group might have been suppressed.

GSEA analysis showed that many ferroptosis-related pathways (including fatty acid metabolism, biological oxidation, glyoxylate metabolism and glycine degradation, peroxisomal lipid metabolism, and mitochondrial fatty acid beta oxidation) were significantly enriched in the low-FHPS group (Figures 5C–H). These results further suggested that ferroptosis was more significant in patients from the low-FHPs group.

To assess the hypoxia status of HCC patients in TCGA cohort, four hypoxia-related gene sets (WINTER_HYPOXIA_METAGENE, WINTER_HYPOXIA_UP, HARRIS_HYPOXIA, and REACTOME_CELLULAR_RESPONSE_TO_HYPOXIA) were selected for GSEA analysis in both groups. As shown in Supplementary Figure S1C, the above four gene sets were significantly enriched in the high-FHPS group, indicating that hypoxia may be more significant in the high FHPS group.

### Analysis of Tumor Immune Cell Infiltration

**FIGURE 6:**
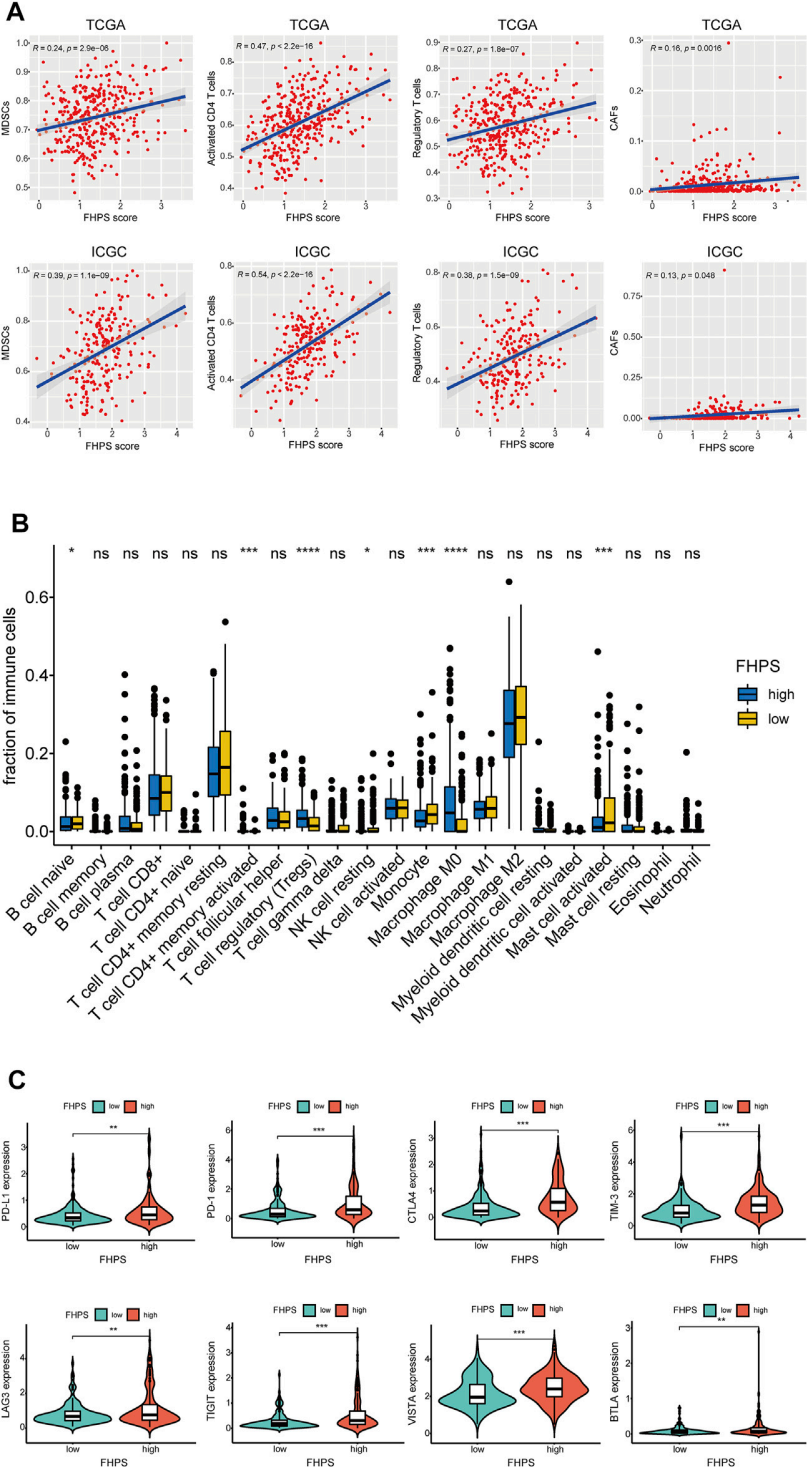
**(A)** ssGSEA and correlation analysis of the FHPS scores and the immune enrichment scores of immune categories in the TCGA and ICGC cohorts. **(B)** Comparison between the fractions of immune cells in the high- and low-FHPS groups of the TCGA cohort *via* the CIBERSORT method. **(C)** Comparison between the expression of several prominent immune checkpoints in the high- and low-FHPS groups of the TCGA cohort. *p < 0.05; **p < 0.01; ***p < 0.001; ****p < 0.0001.

To investigate the relationship between FHPS and tumor immune cell infiltration, ssGSEA was performed in the ICGC cohort and the TCGA cohort. Previous studies have shown that MDSCs have strong immunosuppressive activity and the ability to promote angiogenesis, and are directly involved in promoting the development and progression of tumors. As shown in Figure 6A, FHPS score was significantly positively correlated with the degree of MDSCs infiltration both in the ICGC and TCGA cohorts. In addition, the degree of infiltration of activated CD4^+^ T cells and Tregs were also significantly positively correlated with FHPS score. Moreover, in TCGA and ICGC cohort, the results of EPIC analysis indicated that the degree of infiltration of cancer-associated fibroblasts (CAFs) infiltration were positively correlated with FHPS score (Figure 6A). In TCGA cohort, CIBERSORT analysis further showed that Tregs, Macrophage M0, and activated memory CD4^+^ T cell were more infiltrating in the high-FHPS group, while naive B cell, resting NK cell, Monocyte, and activated Mast cells were more infiltrating in the low-FHPS group (Figure 6B).

Furthermore, we compared the expression of several important immune checkpoints (ICs) between the low-FHPS and high-FHPS groups. As Figure 6C shows, the expression levels of PD-L1, PD-1, TIM-3, CTLA-4, LAG-3, TIGIT, VISTA, and BTLA were significantly higher in the high-FHPS group, suggesting that the high-FHPS group may have better responsiveness to immune checkpoint inhibitors.

### Analysis of Pathway and Process Enrichment

**FIGURE 7:**
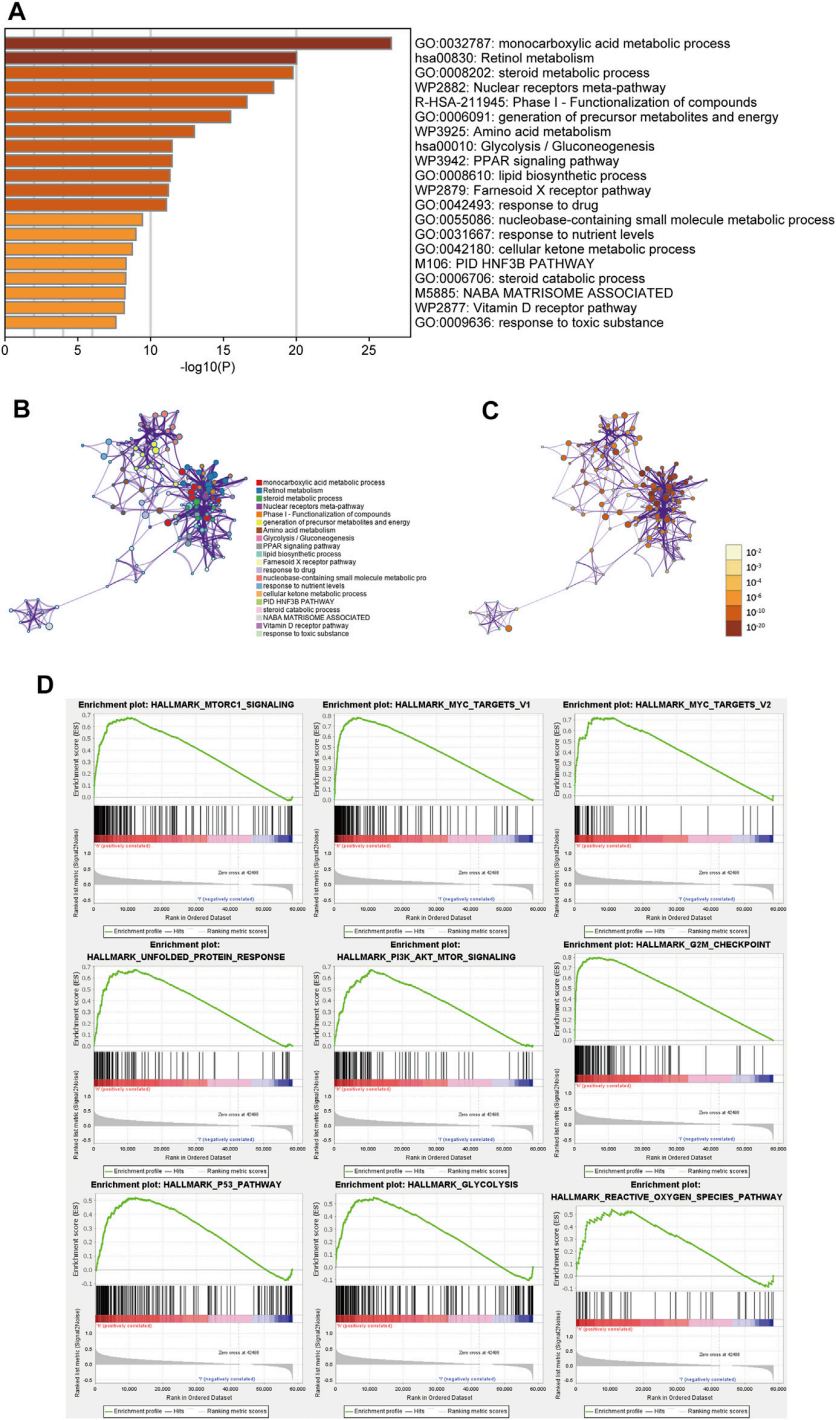
**(A)** Heatmap of enriched terms across the inputted gene list, colored according to *p*-value. Network of enriched terms colored according to **(B)** cluster ID (nodes with the same cluster ID are typically close to each other) and **(C)** *p*-value (terms with more genes tend to have higher *p*-values). **(D)** Gene set enrichment analysis (GSEA) indicating that tumor hallmarks were enriched in the high-FHPS group.

We identified a total of 154 DEGs between the high-FHPS group and the low-FHPS group by using R package “limma”. Then 154 DEGs were uploaded to the metascape website for GO and KEGG analysis. The results showed that these DEGs were significantly enriched in the following terms: monocarboxylic acid metabolic process, steroid metabolic process, nuclear receptors meta-pathway, generation of precursor metabolites and energy, Amino acid metabolism, Glycolysis/Gluconeogenesis, PPAR signaling pathway, and lipid biosynthetic process (Figures 7A–C). GSEA further revealed that several tumor-related hallmarks were enriched in the high-FHPS subgroup, such as MTORC1 signaling, MYC targets V1 and V2, p53 pathway, G2M checkpoint, PIK3-AKT-MTOR signaling, unfolded protein response, glycolysis, and reactive oxygen species pathway (Figure 7D). These results may provide a new perspective and help us to study the potential biological functions and pathways related to FHPS.

### Analysis of Gene Mutation

**FIGURE 8:**
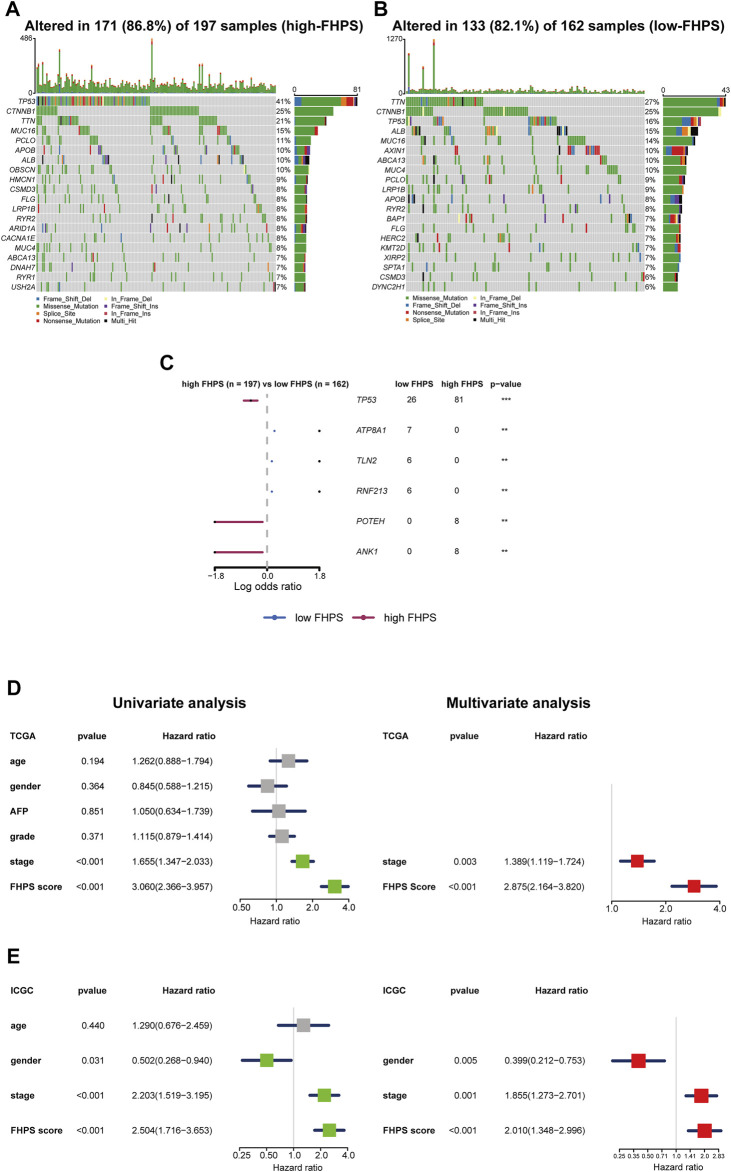
**(A, B)** Oncoplots of the mutated genes in the **(A)** high-FHPS and **(B)** low-FHPS groups of the TCGA cohort. **(C)** Forest plot of the differentially mutated genes between the high- and low-FHPS groups. **(D, E)** Univariate and multivariate analyses revealed that FHPS score was an independent prognostic predictor in the TCGA and ICGC cohorts. *p < 0.05; **p < 0.01; ***p < 0.001; ****p < 0.0001.

To investigate the difference of gene mutation between high-FHPS group and low-FHPS group, we used R package “maftools” to process the simple nucleoside variation data of HCC patients in TCGA cohort. Summary of gene mutation information for the two groups was shown in Supplementary Figures S2A,B. As shown in Figures 8A,B, in the high-FHPS group, the top five genes with mutation frequency were TP53 (41%), CTNNB1 (25%), TTN (21%), MUC16 (15%), and PCLO (11%) while those in the low-FHPS group were TTN (27%), CTNNB1 (25%), TP53 (16%), ALB (15%), and MUC16 (14%). Further analysis showed that TP53, POTEH, and ANK1 with mutation frequencies were higher in the high-FHPS group, while ATP8A1, TLN2, and RNF213 with mutation frequencies were higher in the low-FHPS group (*p* < 0.001) (Figure 8C). In fact, TP53 is a well-researched tumor suppressor gene, and TP53 mutations can lead to the occurrence and progression of a variety of tumors (Villanueva and Hoshida, 2011). Jiang et al. showed that p53 inhibits cystine uptake and sensitizes cells to ferroptosis by repressing expression of SLC7A11 (Jiang et al., 2015). One other study in colorectal cancer showed that TP53 limits ferroptosis by blocking DPP4 activity (Xie et al., 2017). In addition, TP53 mutations can inhibit or induce ferroptosis due to differences in TP53 mutation sites (Jiang et al., 2015; Jennis et al., 2016; Thompson et al., 2020). Notably, TP53 missense mutations can also promote tumor immunosuppression and immune evasion by inhibiting CD8^+^ T cells and enhancing the activation of CAFs (Maddalena et al., 2021). Therefore, the inhibition state of ferroptosis and immunosuppression in the high-FHPS group may be related to the high mutation of TP53.

### FHPS Is an Independent Prognostic Factor for HCC

To verify the independent prognostic value of the FHPS, we performed multivariate and univariate Cox regression analysis on the FHPS score and other clinicopathological features in both cohorts. The results of univariate Cox regression analysis showed that FHPS was a strong risk factor for OS in HCC patients [In TCGA cohort, HR: 3.060, 95% Confidence Interval (CI): 2.366–3.957, *p* < 0.001; In ICGC cohort, HR: 2.504, 95% CI: 1.716–3.653, *p* < 0.001; Figures 8D,E], and multivariate regression analysis showed that FHPS was an independent prognostic factor for HCC patients (In TCGA, HR: 2.875, 95% CI: 2.164–3.820, *p* < 0.001; In ICGC, HR: 2.010, 95% CI: 1.348–2.996, *p* < 0.001). The above results indicated that FHPS was an independent prognostic factor for HCC patients.

### Construction and Validation of Nomogram Base on FHPS

**FIGURE 9:**
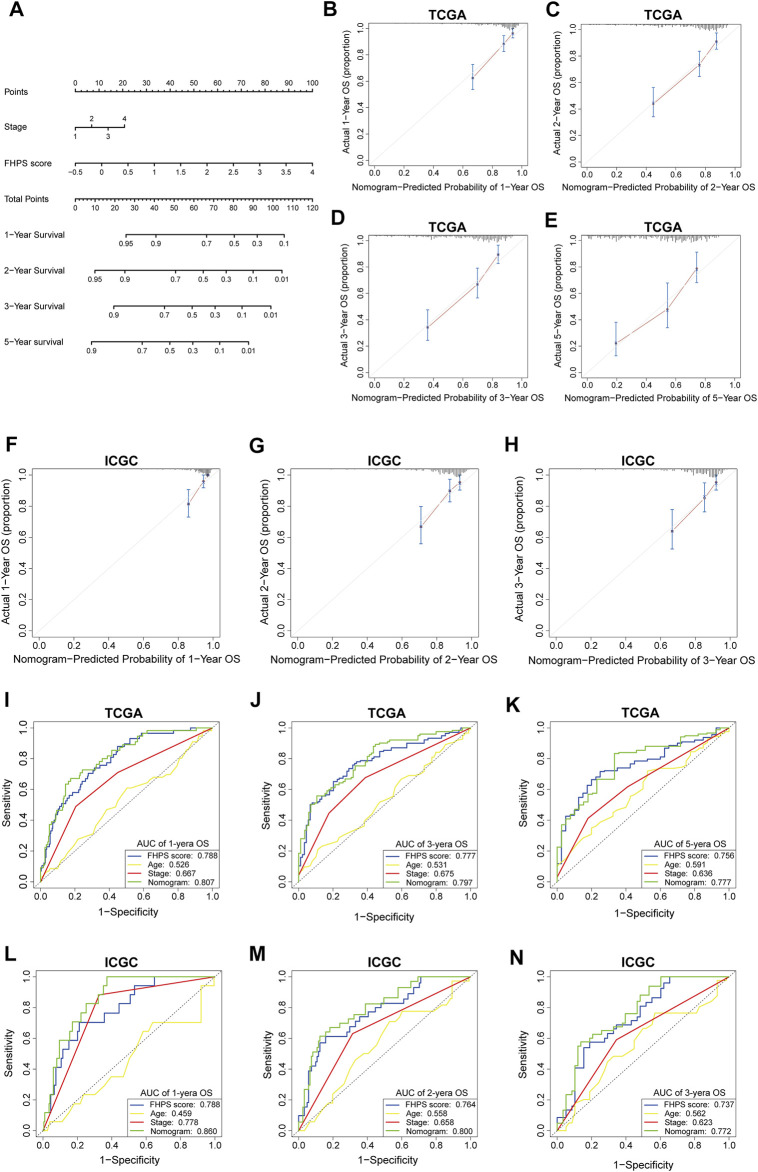
**(A)** Nomogram based on FHPS score, and TNM stage. **(B-E)** Calibration plots of the nomogram for predicting the probability of OS at 1, 2, 3, and 5 years in the TCGA cohort. **(F-H)** Calibration plots of the nomogram for predicting the probability of OS at 1, 2, and 3 years in the ICGC cohort. **(I–K)** ROC curves for the nomogram, FHPS score, age, and TNM stage in the TCGA cohort (for predicting 1, 3, and 5-years OS). **(L–N)** ROC curves for the nomogram, FHPS score, age, and TNM stage in the ICGC cohort (for predicting 1, 2, and 3-years OS).

To better apply FHPS to the clinic, we developed a nomogram based on FHPS and other independent prognostic factors (TNM stage) in TCGA cohort (Figure 9A), which was validated in ICGC cohort. The C-index was 0.734 in the TCGA training cohort and 0.764 in the ICGC validation cohort. Calibration curves showed that the predicted rates were highly concordant with the actual rates for 1-, 2-, 3-, and 5-years survival in both cohorts (Figures 9B–H). Moreover, ROC curves showed that the prognostic predictive ability of the nomogram in HCC patients was superior to other factors (Including FHPS score, gender, age and TNM stage). The AUCs of 1-, 3-, and 5-years reached 0.807, 0.797, and 0.777 in TCGA cohort (Figures 9I–K) and 0.860, 0.800, and 0.772 (1-, 2- and 3-years) in ICGC cohort (Figures 9L–N). These results indicated that our nomogram based on FHPS score and TNM stage has a strong and stable ability to predict the OS of HCC patients.

### Differences in the Protein Expression of 10 Prognostic FRGs or HRGs Between in Normal Liver Tissue and HCC Tissue

**FIGURE 10:**
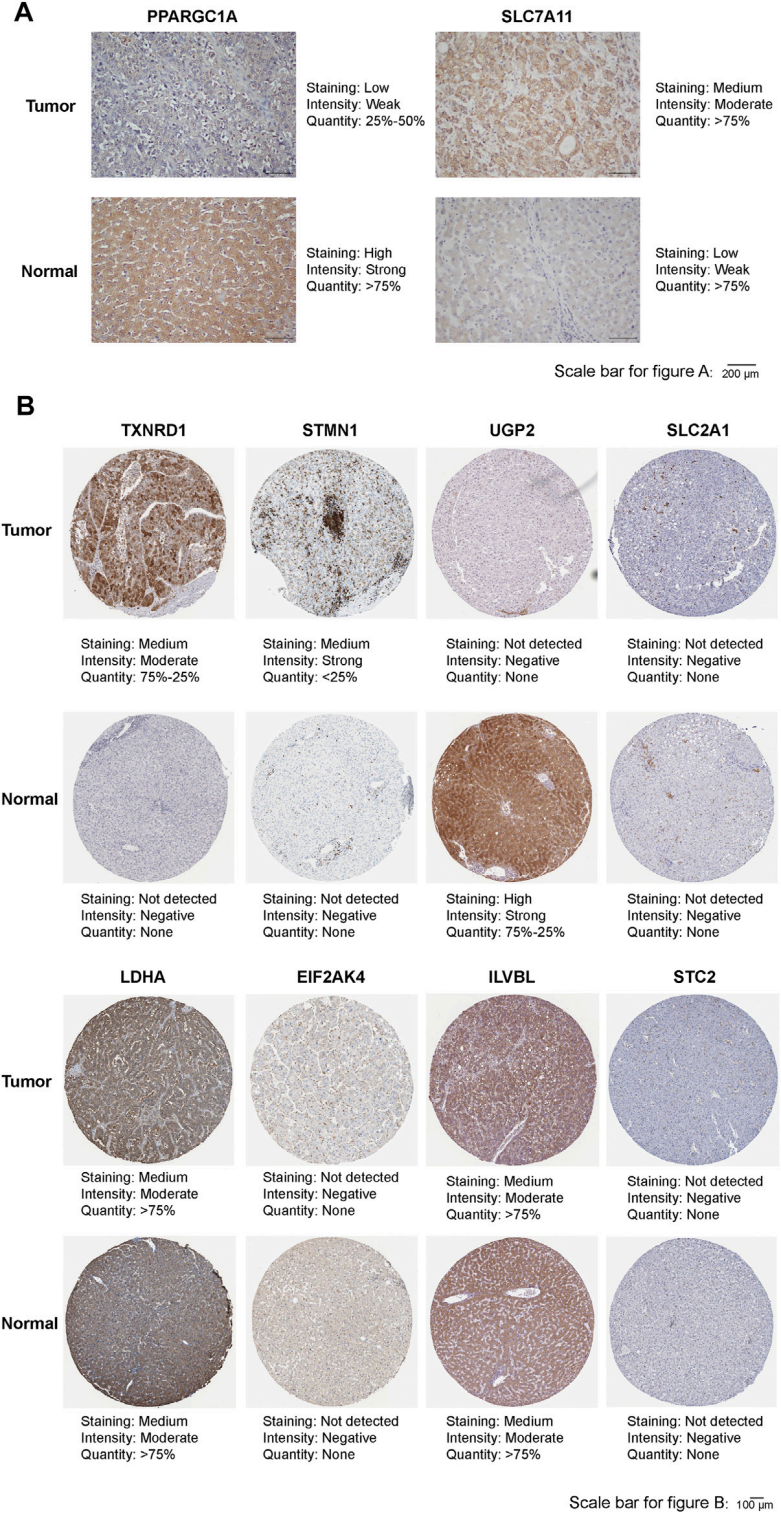
**(A)** Representative immunohistochemistry images of PPARGC1A and SLC7A11 in HCC tissues and adjacent normal tissues. **(B)** Representative immunohistochemistry images of TXNRD1, STMN1, UGP2, SLC2A1, LDHA, EIF2AK4, ILVBL, and STC2 in HCC tissues and normal tissues.

As shown in Figure 10A, the protein expression of SLC7A11 was higher in HCC tissues, whereas that of PPARGC1A was higher in adjacent normal tissues by immunohistochemical staining. The immunohistochemical staining images of the remaining eight prognostic FRGs or HRGs from HPA showed that the protein expression of TXNRD1 and STMN1 were higher in HCC tissues, that of UGP2 was higher in normal liver tissues, while the protein expression of SLC2A1, LDHA, EIF2AK4, ILVBL, and STC2 showed no significant difference between normal liver tissues and HCC tissues (Figure 10B). Moreover, we compared the expression of PPARGC1A and SLC7A11 in HCC tissues of different WHO grade and TNM stage, and the results showed that the protein expression of SLC7A11 was higher in advanced stage and high-grade HCC tissues, while protein expression of PPARGC1A was higher in early stage and low-grade HCC tissues (Supplementary Figure S3). In addition, due to the lack of WHO grade and TNM stage information in HPA database, immunohistochemical staining images of the remaining eight genes could not be compared between different WHO grade and TNM stage HCC tissues.

## Discussion

Ferroptosis, as a unique programmed cell death modality, has been found to be closely related to the development and occurrence of HCC. Lin et al. and Hollande et al. found that sorafenib and immunotherapy can exert anti-tumor effects by inducing ferroptosis in HCC cells (Louandre et al., 2013; Wang et al., 2019). A recent *in vitro* study showed that artesunate greatly enhanced the anticancer effects of low-dose sorafenib in Huh7, snu-449, and snu-182 HCC cell lines, and this synergistic effect was mediated by induction of ferroptosis in HCC cells (Li et al., 2021). Because of the heterogeneity between tumor cells, they have different sensitivities to ferroptosis (Friedmann Angeli et al., 2019), which may affect the responsiveness of sorafenib and immunotherapy. With the deepening of the research on tumor microenvironment, hypoxia signaling pathway has been found to be extensively involved in the transformation and growth of HCC and to inhibit anti-tumor therapy by regulating proliferation, mutation, angiogenesis, metastasis, and apoptosis (von Marschall et al., 2001; Gwak et al., 2005; Piret et al., 2005). In HCC, treatment with sorafenib would lead to a hypoxic tumor microenvironment [Sorafenib-induced hypoxia (SIH)] due to pericyte depletion and reduction of tumor vessels, while SIH further promoted the accumulation and stabilization of HIF-1α and HIF-2α, leading to oncogene transcription and angiogenesis, which promoted the resistance of HCC cells to sorafenib (Cooke et al., 2012; Dong et al., 2018; Song et al., 2019). In addition, hypoxia can also promote drug resistance in various tumors through multiple pathways, including overexpression of drug efflux proteins, apoptosis inhibition, autophagy induction, DNA damage inhibition, and mitochondrial activity (van Loo et al., 2002; Fulda et al., 2010; Madan et al., 2012; Hu et al., 2015; He et al., 2017). However, at the present time, there is no high-throughput study to explore the potential link between ferroptosis and hypoxia, as well as their combined effect on the prognosis of HCC.

In this study, we included 370 patients in TCGA database and 231 patients in ICGC database. Using the known FRGs and HRGs, we constructed a signature (FHPS) for predicting the prognosis of HCC patients. According to the best optimal cut-off value, the patients were divided into low-FHPS group and high-FHPS group. Excitingly, we found consistent results in the TCGA training cohort, as well as in the independent ICGC validation cohort, that patients in the high-FHPS group had poor tumor differentiation, advanced TNM staging, shorter overall survival time, and higher mortality. It means that we can use this signature to distinguish patients with poor prognosis and late stage at the time of diagnosis, and then implement individualized treatment plans for these patients, such as increasing the follow-up frequency of patients with high FHPS score to monitor the progress of the disease. To better apply FHPS to clinic, we developed a nomogram based on FHPS score and TNM stage to predict the survival rates (1-, 2-, 3-, and 5-yeras) of HCC patients. Calibration Curves, ROC Curves, and C-index show that this Nomogram has a strong ability to predict the prognosis of HCC patients, which may be beneficial for making personalized clinical decisions for HCC patients. In addition, based on GSEA and a set of well-researched SOFs, we found that patients in the high-FHPS group showed ferroptosis suppressive status and high hypoxia status, which may be critical factors for the poor prognosis of patients in the high-FHPS group.

Then, we further explored the correlation between FHPS and TIME, and the results exhibited that the high-FHPS group showed significant immunosuppression. ssGSEA revealed a significant positive correlation between the FHPS score and the infiltration of MDSCs. Several studies have reported that MDSCs exert immunosuppressive effects through multiple pathways, such as inducing the expansion and differentiation of Tregs and limiting the polarization of NKs, DCs, and macrophages to M2-phenotype (Chiu et al., 2016; Sun et al., 2018; Guha et al., 2019; Lu et al., 2019). Hypoxia can promote the secretion of CCL26 by tumor cells to recruit MDSCs, which is the main driver of MDSCs accumulation in tumor immune microenvironment (Chiu et al., 2016; Chiu et al., 2017). Further analysis also revealed a positive correlation between the FHPS score and the infiltration of Tregs as well as CAFs. Tregs can limit a variety of immune pathways including autoimmunity, immunity to pathogenic microorganisms, allografts, and tumors. And tumor-associated Tregs also directly promote tumor immune evasion through several contact-independent and contact-dependent mechanisms (Sakaguchi et al., 2008; Sakaguchi et al., 2010). Hypoxia promotes tumor cells to express a large amount of CCL28 receptors, which recruit Tregs into tumor tissues and interact with CCR10 on Tregs surface to exert immunosuppressive effect (Facciabene et al., 2011). CAFs promote tumor proliferation, invasion, and metastasis by secreting various cytokines and growth factors (Terada et al., 1996; Tomasek et al., 2002). In addition, CAFs have also been found to play an immunosuppressive role in HCC by inducing NK cells to transform into inactive phenotypes, making NKs cells unresponsive in tumors (Li et al., 2012). Notably, FHPS score was also found to be positively correlated with the CD4^+^ T cell, which is generally considered as a tumor-toxic T cell (Jin et al., 2018). However, Astrid et al. found that hypoxia affects the transformation of CD4^+^ T cells into TH1 effector cells and reduces the production of IFN-γ, which plays an important role in an effective antitumor response (Westendorf et al., 2017). Indeed, in our study, patients in the high-FHPS group were found to have a high hypoxia state, suggesting that the anti-tumor effect of significantly infiltrated CD4^+^ T cells may be limited due to hypoxia, which further promotes an immunosuppressive state. Consistent with this, CIBERSORT analysis further confirmed that Tregs were significantly infiltrated in the high-FHPS group. In conclusion, high FHPS score is associated with immunosuppression of HCC, in which hypoxia may play a critical role. Then, we compared the expression of several important ICs between the low-FHPS and high-FHPS groups. The results showed that the expression of PD-L1, PD-1, TIM-3, CTLA-4, LAG-3, VISTA, TIGIT, and BTLA were significantly up-regulated in the high-FHPS group. This suggests that patients in the high-FHPS group may benefit from treatment with ICIs.

Among the ten genes in the FHPS that we constructed, as the target gene of HIF-1α, SLC2A1 is involved in the formation of hypoxia in tumor microenvironment (Lequeux et al., 2021). Meanwhile, SLC2A1 mediated glucose transport promotes glycolysis, accelerates fatty acid synthesis, and ultimately promotes lipid peroxidation dependent ferroptosis (Song et al., 2021). In addition, SLC2A1 was found to be upregulated in a variety of tumors, including liver cancer, breast cancer, lung cancer, and so on (Wachi et al., 2005; Amann et al., 2009). Like SLC2A1, LDHA was found to increase expression during the transition from precancerous lesions to invasive carcinomas, and multiple studies have confirmed that LDHA is closely related to tumor initiation and progression (Koukourakis et al., 2003; Fantin et al., 2006; Xie et al., 2009; Ooi et al., 2014). Moreover, Cui et al. found that HIF-1α and HIF-2α can induce LDHA expression in anoxic microenvironment (Cui et al., 2017). SLC7A11, a well-researched SOF, inhibits ferroptosis by promoting glutathione synthesis and reducing lipid peroxidation (Dixon et al., 2012). Previous studies have shown that the upregulation of SLC7A11 is associated with poor prognosis of HCC, while the downregulation of SLC7A11 can inhibit the growth of HCC (Guo et al., 2011; Kinoshita et al., 2013). In addition, He et al. showed that SLC7A11 can up-regulate the expression of PD-L1 and CSF1, and promote the infiltration of TAMs and MDSCs in HCC, as well as the metastasis of HCC (He et al., 2021). STC2 is the target gene of HIF-1 and has been proved to be a prognostic marker of ovarian cancer, breast cancer, and kidney cancer (Chang et al., 2003; Buckanovich et al., 2007; Esseghir et al., 2007). In addition, the study of Alice et al. further suggested that STC2 is a positive regulator of tumor progression under hypoxia (Law and Wong, 2010). STMN1 has been found to be overexpressed in a variety of tumors, correlates with poor prognosis, and has been implicated as an important stimulator of metastasis in liver cancer (Cheng et al., 2008; Zheng et al., 2015; Bai et al., 2017). In addition, ZHANG et al. found that STMN1 can regulate glutathione production via PSAT1 to exert a ferroptosis inhibitory effect (Zhang et al., 2019). TXNRD1 was found to be overexpressed in HCC and associated with poor survival and advanced tumor staging (Fu et al., 2017). PPARGC1A is a transcription coactivator that binds with a variety of transcription factors to regulate the expression of target genes (Puigserver and Spiegelman, 2003). The study of Wang et al. suggested that overexpressed miR-93-5p enhanced the proliferation capacity of HCC by inhibiting the expression of PPARGC1A. A previous study suggested that low UGP2 expression was independently associated with poor prognosis of HCC (Hu et al., 2020). ILVBL is the major 2-OH acyl-CoA lyase involved in the cleavage reaction in the fatty acid α-oxidation of the phytosphingosine degradation pathway (Kitamura et al., 2017). Current studies have shown that EIF2AK4 is a key gene in the progression of pulmonary veno-occlusive disease (Eyries et al., 2014). However, the role of EIF2AK4 and ILVBL in tumors has not been clearly established, and our study suggests that EIF2AK4 and ILVBL were prognostic protective factors for HCC.

At present, some studies have used FRGs or HRGs to build prognostic models for HCC (Liang et al., 2020; Bai et al., 2021), but considering the heterogeneity of HCC and the complexity of tumor microenvironment, the prognostic methods of ferroptosis or hypoxia alone lack high specificity and sensitivity. In this study, we considered the impact of both ferroptosis and hypoxia on the prognosis of HCC, using FRGs and HRGs together to construct an FHPS, so it has a stronger ability to predict prognosis. The establishment of nomogram makes FHPS more convenient for clinical application. In addition, FHPS can also distinguish ferroptosis, hypoxia, and immunosuppression status of HCC, which provides a new idea for individualized treatment of HCC patients. At the same time, this study also has the following limitations: 1. As a retrospective study, some bias is inevitable, and a multi-center prospective cohort is required to verify this signature in the future; 2. The potential link between ferroptosis and hypoxia needs to be further explored *in vivo* and *in vitro* studies.

## Data Availability

The original contributions presented in the study are included in the article/Supplementary Materials, further inquiries can be directed to the corresponding authors.

## Footnotes

1
https://portal.gdc.cancer.gov/

2
https://dcc.icgc.org/

3
http://www.gsea-msigdb.org/gsea/msigdb

4
https://CRAN.R-project.org/package=survminer

5
https://metascape.org

6
https://www.proteinatlas.org/

## Supplementary Material

Supplementary Figure 1
**(A,B)** Comparison of the expressions of the drivers of ferroptosis between the high- and low-FHPS groups of the **(A)** TCGA and **(B)** ICGC cohorts. **(C)** GSEA (Hypoxia-related gene sets) of the high- and low-FHPS groups in the TCGA cohorts. **p* < 0.05; ***p* < 0.01; ****p* < 0.001; *****p* < 0.0001

Supplementary Figure 2
**(A,B)** Summary of gene mutation information in the low-FHPS group **(A)** and high-FHPS group **(B)** in the TCGA cohort.

Supplementary Figure 3 Representative immunohistochemistry images of PPARGC1A and SLC7A11 in different WHO grade and TNM stage HCC tissues.

Supplementary Table 1 The clinicopathological information of HCC and normal liver tissues used for immunohistochemical staining.
