# Supplementary figures and images for: Construction and Validation of a Combined Ferroptosis and Hypoxia Prognostic Signature for Hepatocellular Carcinoma
Source: Front Mol Biosci. 2021 Dec 17;8:809672. doi: 10.3389/fmolb.2021.809672 (PMC8719198; doi:10.3389/fmolb.2021.809672)

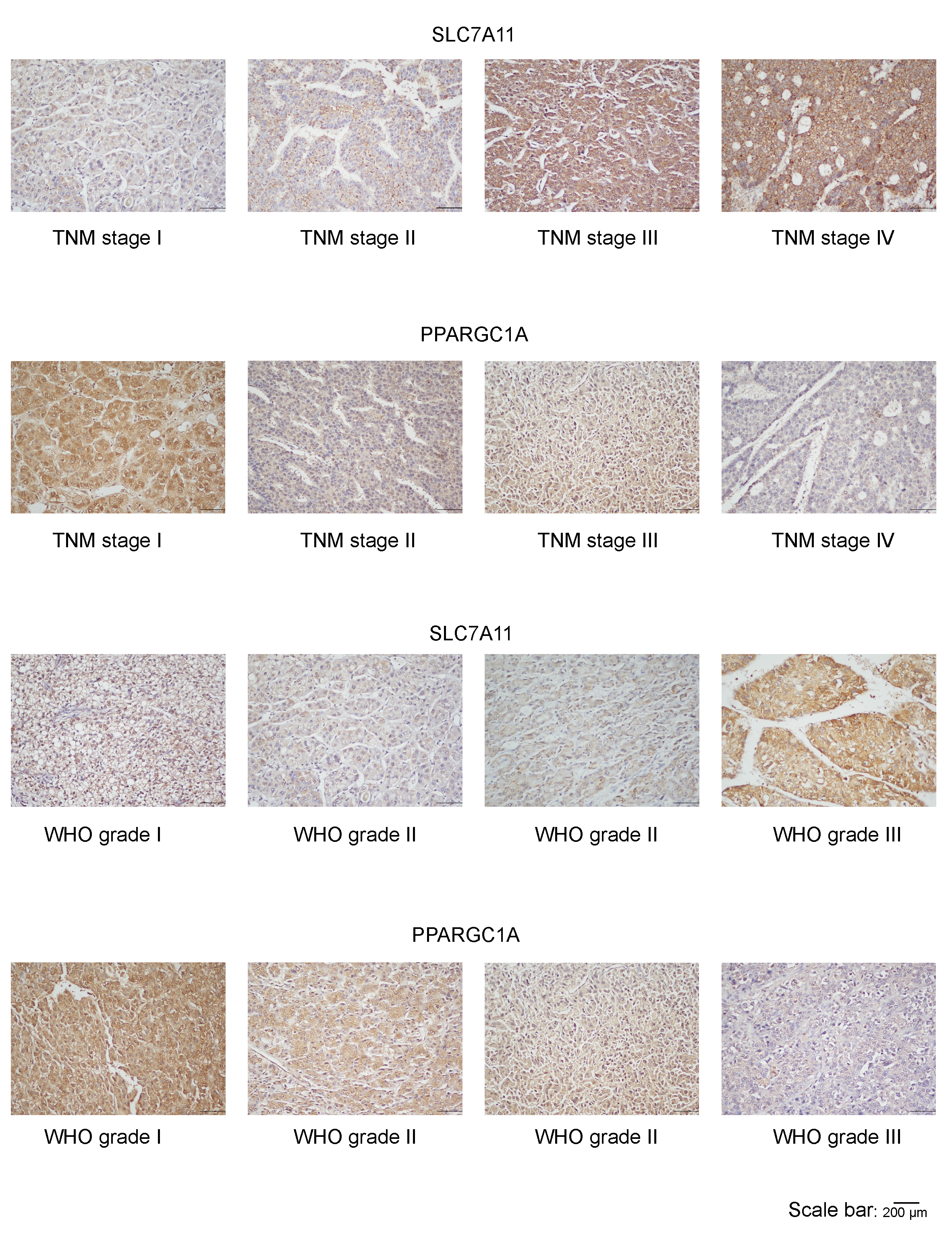

Supplement: Supplementary file 1 [file Image3.TIF]

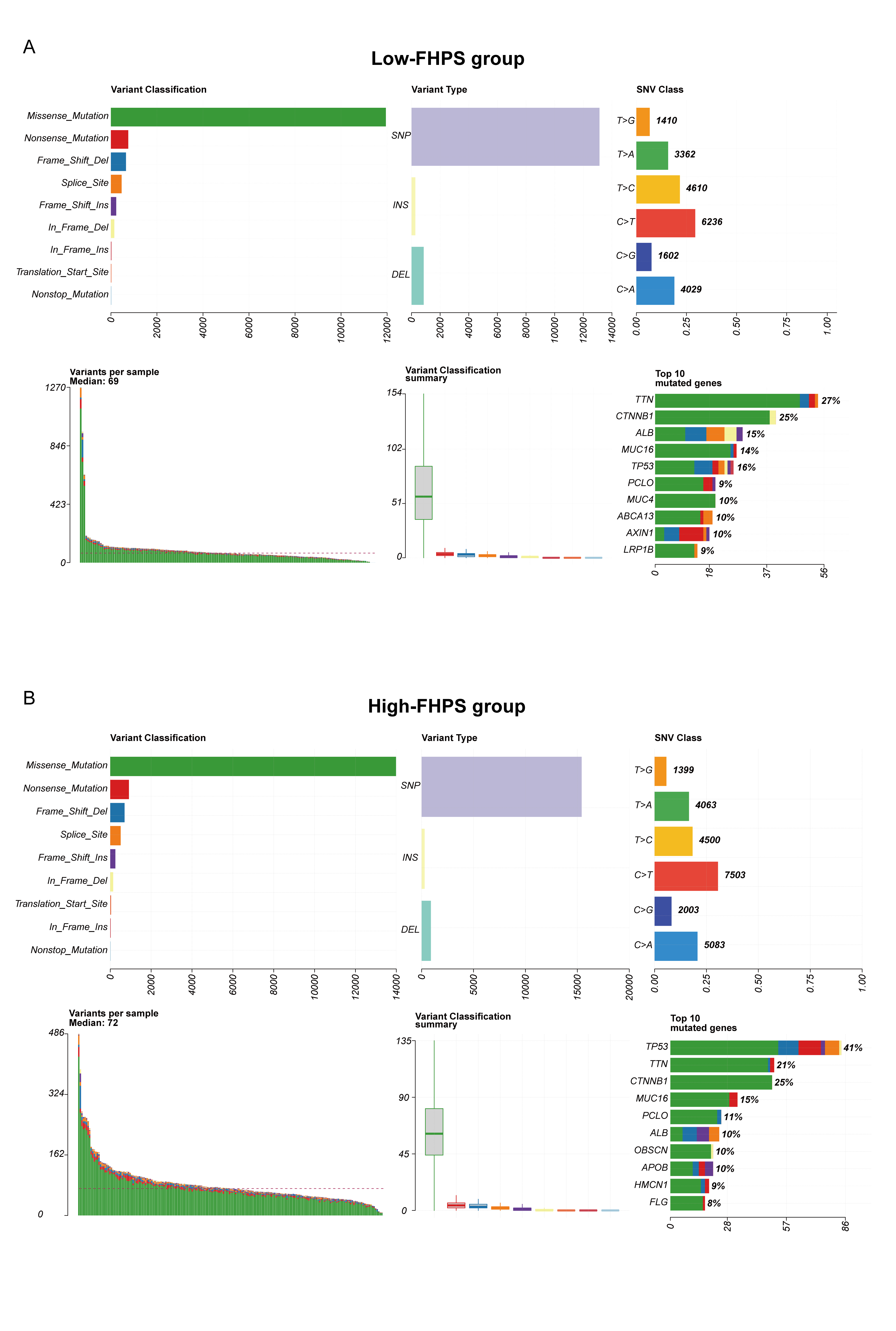

Supplement: Supplementary file 2 [file Image2.TIF]

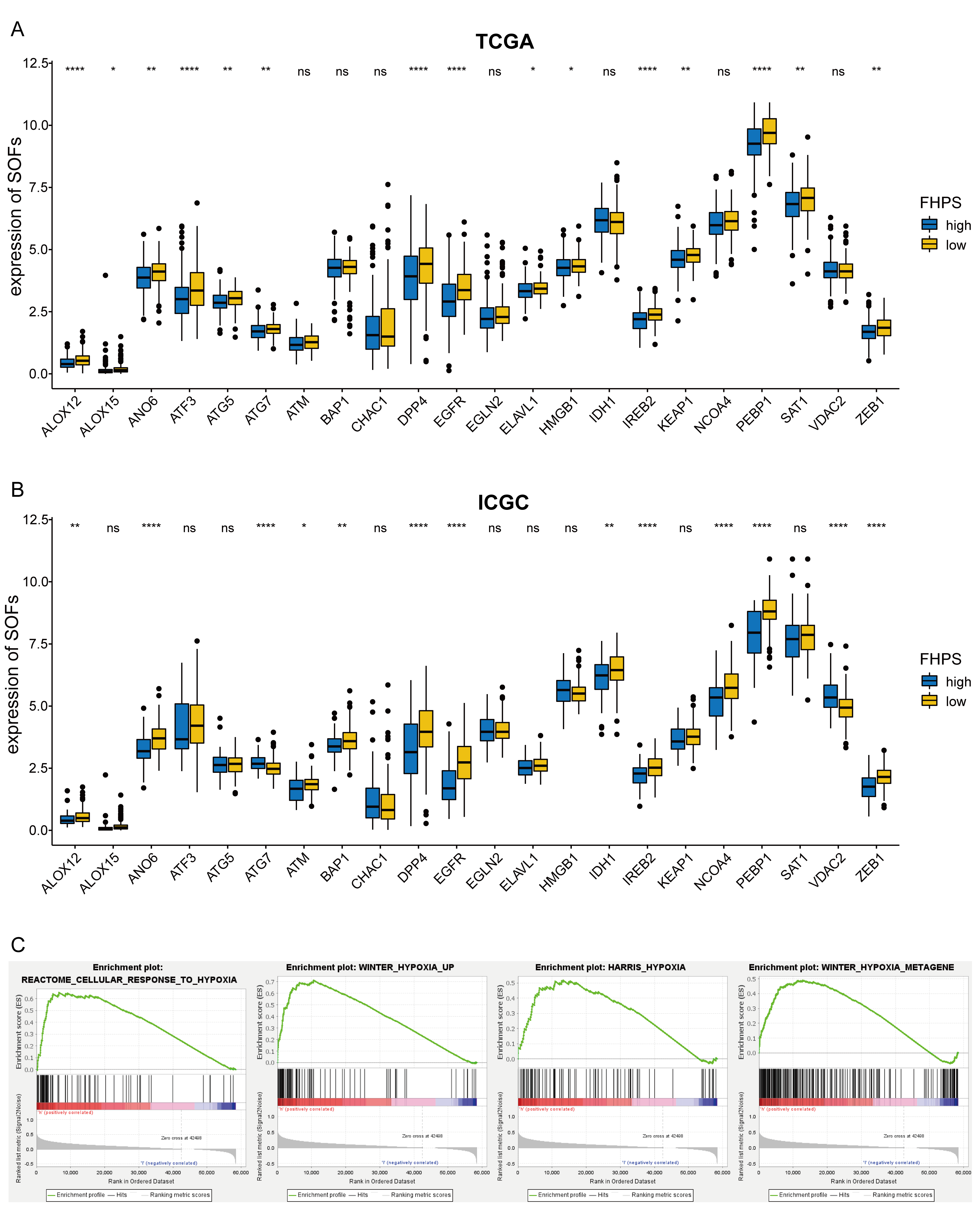

Supplement: Supplementary file 3 [file Image1.TIF]
